# Supplementary material for: Development and Validation of an HPLC Method for the Quantitative Analysis of Bromophenolic Compounds in the Red Alga Vertebrata lanosa
Source: Mar Drugs. 2019 Nov 29;17(12):675. doi: 10.3390/md17120675 (PMC6950000; doi:10.3390/md17120675)
Supplement: Supplementary file 1 [file marinedrugs-17-00675-s001.pdf]

Supplementary material

Development and validation of an HPLC method for the quantitative analysis of bromophenolic compounds in the red alga Vertebrata lanosa

Stefanie Hofer [1], Anja Hartmann [1*], Maria Orfanoudaki [1], Hieu Ngoc Nguyen [1], Markus Nagl[3], Ulf Karsten [2], Svenja Heesch [2] and Markus Ganzera [1]

[1] Department of Pharmacognosy, University of Innsbruck, Innrain 80-82, Innsbruck, 6020, Austria; e-mails: stefanie.hofer@uibk.ac.at (S.H.); anja.hartmann@uibk.ac.at (A.H.); maria.orfanoudaki@uibk.ac.at (M.O.); hieunguyen@snu.ac.kr (H.N.N.); markus.ganzera@uibk.ac.at (M.G.)

[2] Institute of Biological Sciences, Applied Ecology & Phycology, University of Rostock, Albert-Einstein-Str. 3, Rostock, 18059, Germany; emails: ulf.karsten@uni-rostock.de (U.K.); svenja.heesch@uni-rostock.de (S.H.)

[3] Institute of Hygiene and Medical Microbiology, Medical University of Innsbruck, Schöpfstraße 41, Innsbruck, 6020, Austria; e-mail: m.nagl@i-med.ac.at (M.N.)

* Correspondence: anja.hartmann@uibk.ac.at

Received: date; Accepted: date; Published: date

Content:

Table S1. NMR shift values for Compounds 2-7.

Table S2. Screened algae

Figure S1. HPLC separation of 7 standards with different gradients.

Figure S2. HPLC-MS analysis of the methanolic Vertebrata lanosa extract.

Figure S3. HPLC analysis after sample enrichment on an SPE cartridge.

Figure S4. 1H-NMR and 13C-NMR spectra of the new compound.

Figure S5. HMBC, COSY and HSQC spectra of the new compound.

	Methylrhodomelol **(2)**		Lanosol **(3)**		Lanosol methyl ether **(4)**	
	^{13}C	^{1}H	^{13}C	^{1}H	^{13}C	^{1}H
1′	128.0	-	134.3	-	131.0	-
2′	117.9	-	114.1	-	115.3	-
3′	113.6	-	114.2	-	114.3	-
4′	144.9	-	144.8	-	145.3	-
5′	145.8	-	146.4	-	146.3	-
6′	118.7	6.93 (s)	114.9	7.01 (s)	115.8	6.92 (s)
7′	39.0	3.33 (d)	65.7	4.55 (d)	75.9	4.43 (s)
1	-	-	-	-	58.5	3.40 (s)
2	173.2	-	-	-	-	-
3	84.0	-	-	-	-	-
3a	110.5	-				
4	-	-	-	-	-	-
5	76.9	4.25/4.12 (m)	-	-	-	-
6	74.8	4.40 (m)	-	-	-	-
6a	88.9	4.61 (d)	-	-	-	-
7	54.6	3.58 (m)				

	3-Bromo-4-(2,3.dibromo-4,5-dihydroxybenzyl)-5-methoxymethylpyrocatechol **(5)**		5-((2,3-Dibromo-4,5-dihydroxybenzyloxy)methyl)-3,4-dibromobenzene-1,2-diol **(6)**		2,2‘,3,3‘-Tetrabromo-4,4‘,5,5‘-tetrahydroxydiphenylmethane **(7)**	
	^{13}C	^{1}H	^{13}C	^{1}H	^{13}C	^{1}H
1′	127.5	-	129.1	-	133.1	-
2′	113.1	-	113.3	-	117.4	-
3′	142.8	-	113.2	-	115.0	-
4′	144.4	-	145.1	-	145.0	-
5′	115.6	6.86 (s)	144.0	-	147.0	-
6′	128.5	-	114.9	7.01 (s)	117.2	6.47 (s)
7′	72.1	4.12 (s)	72.1	4.51 (s)	-	-
1″	57.4	3.18 (s)	-	-	-	-
1	130.4	-	129.1	-	133.1	-
2	114.6	-	113.3	-	117.4	-
3	114.3	-	113.2	-	115.0	-
4	142.8	-	145.1	-	145.0	-
5	145.1	-	144.0	-	147.0	-
6	113.9	6.02 (s)	114.9	7.01 (s)	117.2	6.47 (s)
7	38.3	3.97 (s)	72.1	4.51 (s)	45.9	4.03 (s)

Table S1. NMR shift values for Compounds **2-7** in MeOD (**2-4**) or DMSO (**5-7**); the spectra were recorded on a 600 MHz NMR instrument.

	Name of alga/lichen
1	*Jania rubens*
2	*Colpomenia peregrina*
3	*Dictyota dichotoma*
4	*Grateloupia turuturu*
5	*Calliblepharis jubata*
6	*Lichina pygmea*
7	*Ceramium sp.*
8	*Laminaria ochroleuca*
9	*Ulva lactuca*
10	*Cladophora sp.*
11	*Pelvetia canaliculata*
12	*Ulva sp.*
13	*Gracilaria gracilis*
14	*Fucus spiralis*
15	*Saccharina latissima*
16	*Himanthalia elongata*
17	*Osmundea sp.*
18	*Mastocarpus stellatus*
19	*Ascophyllum nodosum*
20	*Chondrus crispus*

Table S2. Algae and one lichen that were screened for the presence of the 7 bromophenols; all collected 2018, Roscoff, Brittany, 48.727559 ºN; 3.987924 ºW, collected and identified by U.Karsten and S.Heesch University of Rostock.

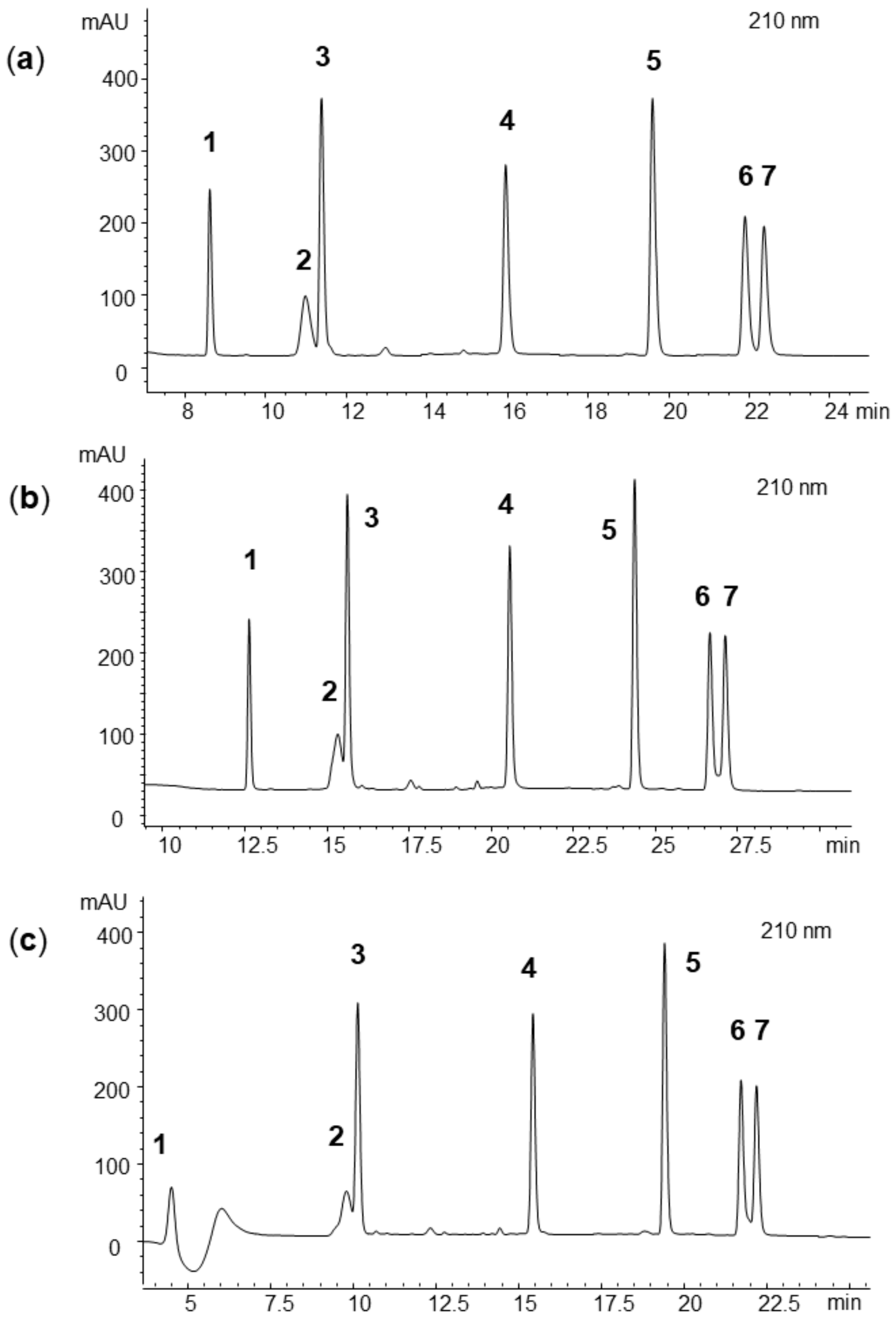

Figure S1. (**a**) HPLC separation of 7 standards under optimized conditions with a gradient of 2% B at 0 min, 20% B at 0.1 min, 50% B at 15 min, 70% B at 35 min (**b**) Poorer HPLC separation of the standards if 2% B was left for 5 min (**c**) Starting conditions of 20 % B resulted negatively on the separation of peak 2 and 3.

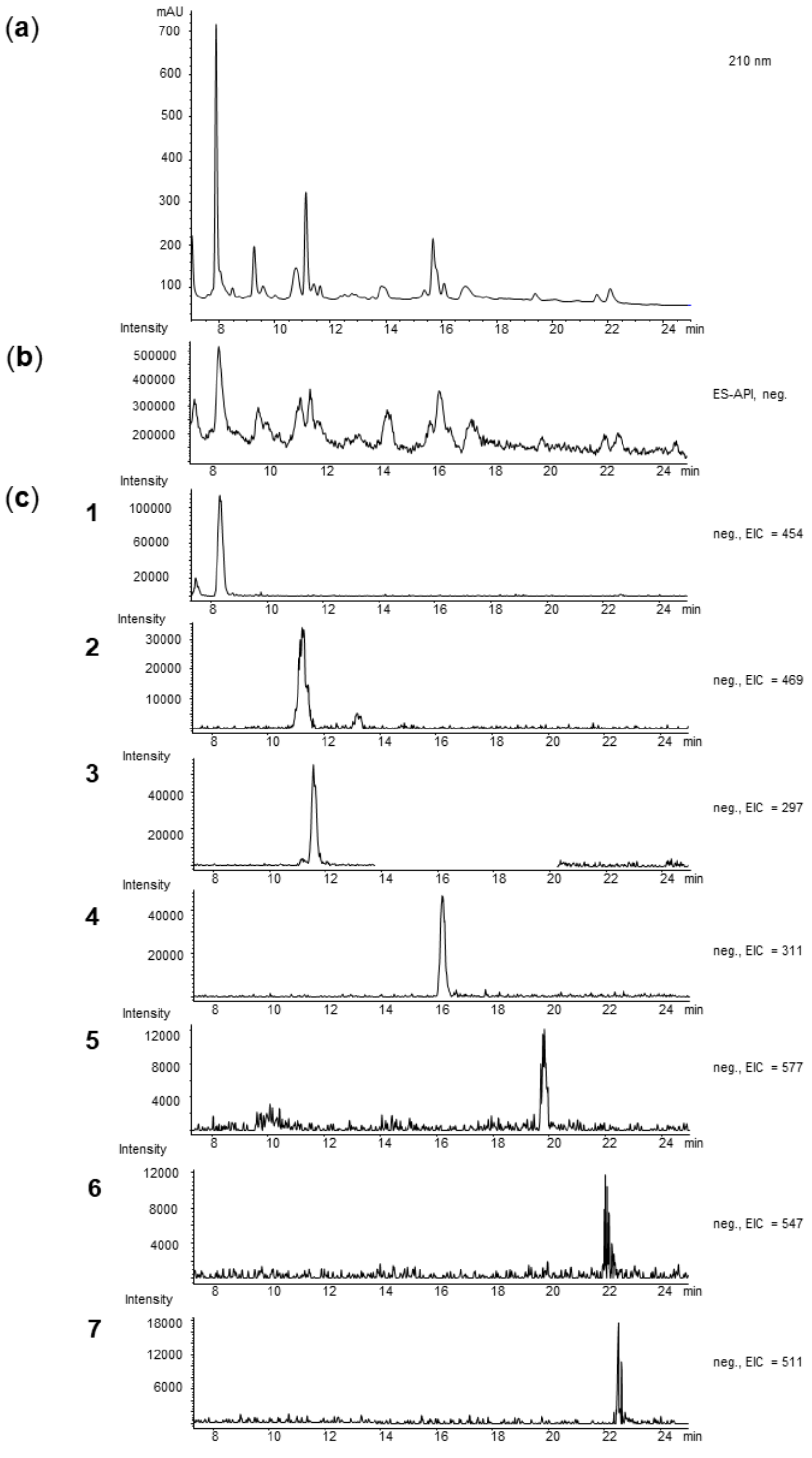

Figure S2. HPLC-MS analysis of the methanolic *Vertebrata lanosa* extract under optimized conditions with a gradient of 2% B at 0 min, 20% B at 0.1 min, 50% B at 15 min, 70% B at 35 min using water and acetonitrile each containing 0.1% formic acid as mobile phase; **(a)** detection: 210 nm (**b**) mass detection by applying ESI-API in negative mode (**c**) extracted ion chromatograms of the 7 isolated bromophenols.

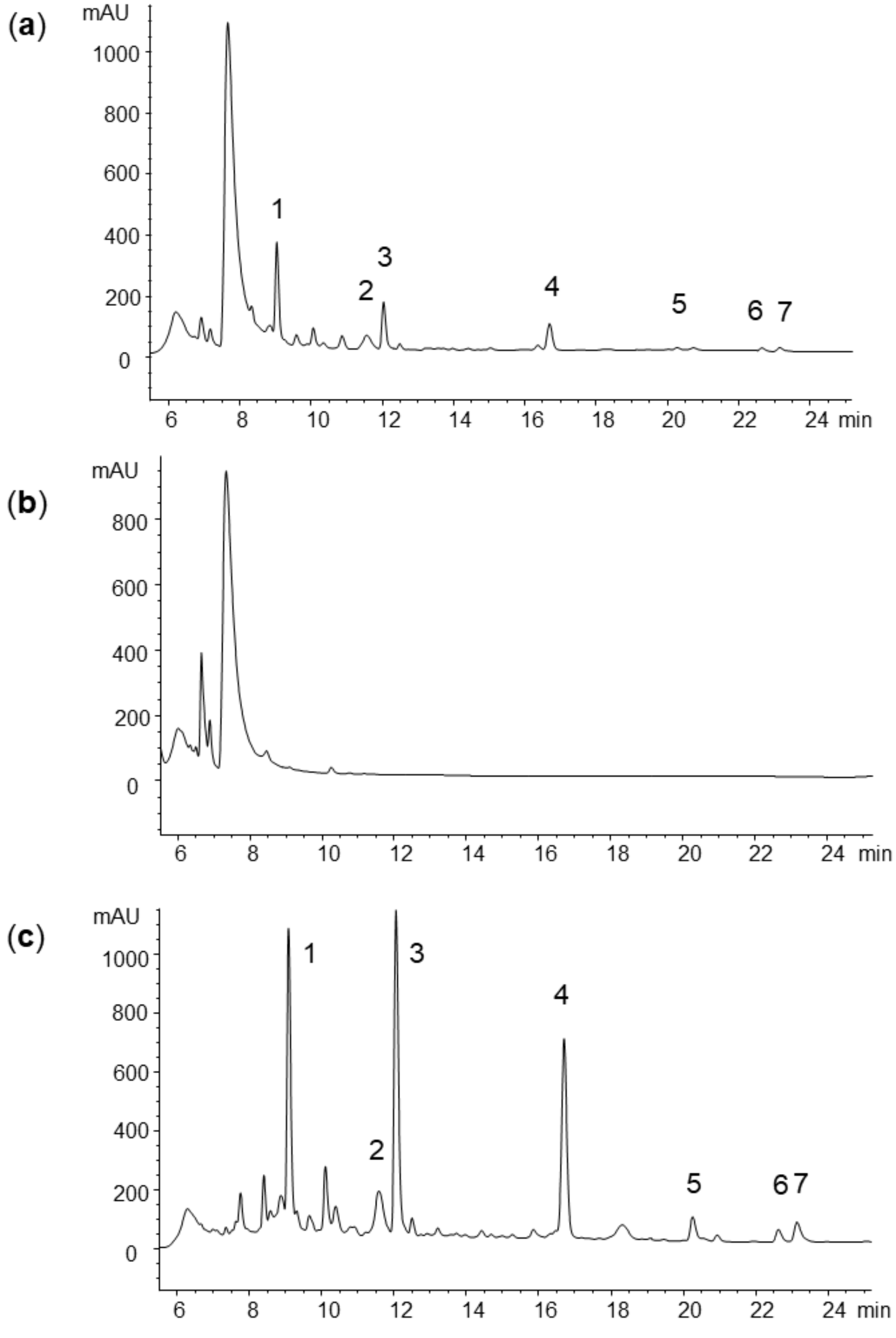

Figure S3. (**a**) Crude methanolic extract of *Vertebrata lanosa;* (**b**) chromatogram of the impurities that were selectively removed; (**c**) sample after enrichment on an SPE cartridge.

(a)

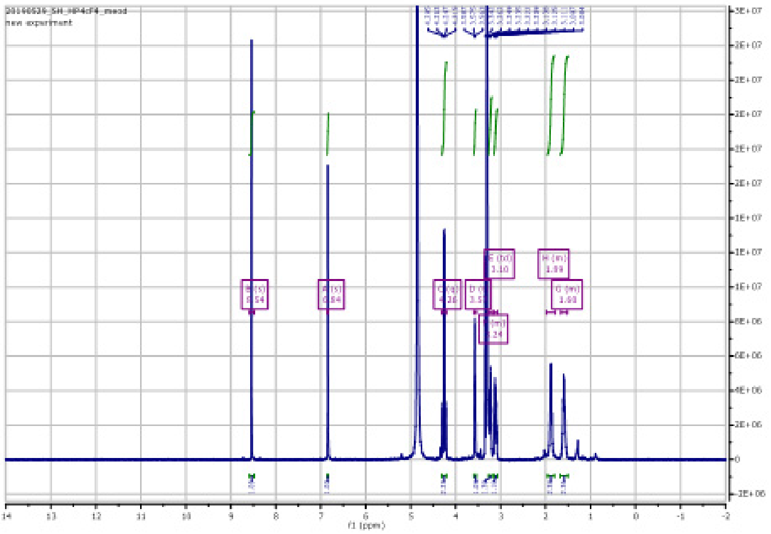

(b)

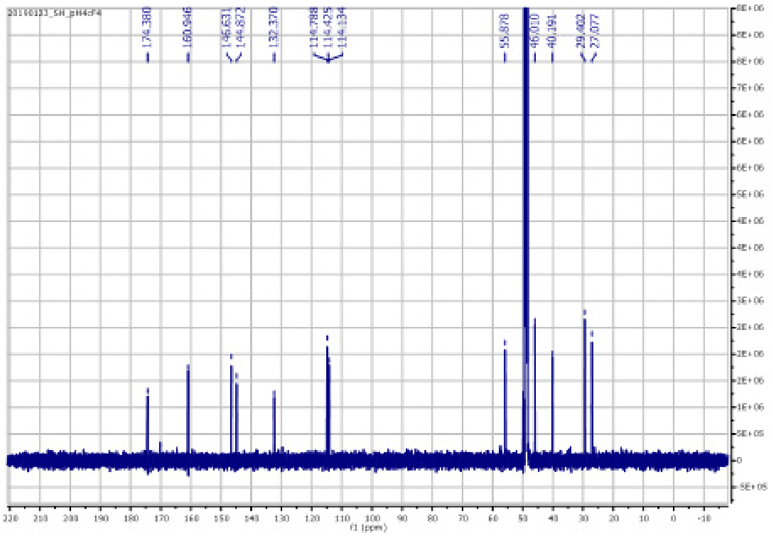

Figure S4. (a) 1H-NMR and (**b**) 13C-NMR spectra of the new compound, recorded in MeOD on a 600 MHz NMR instrument.

(a)

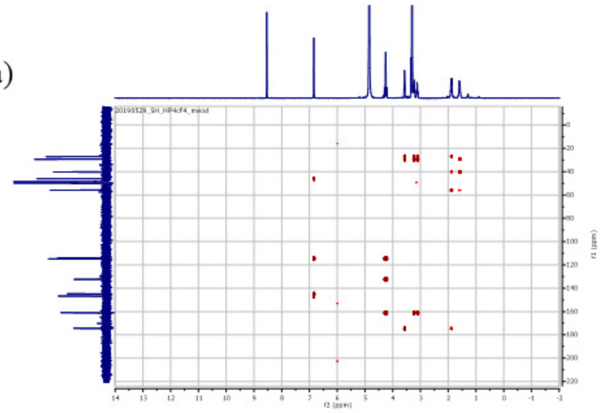

(b)

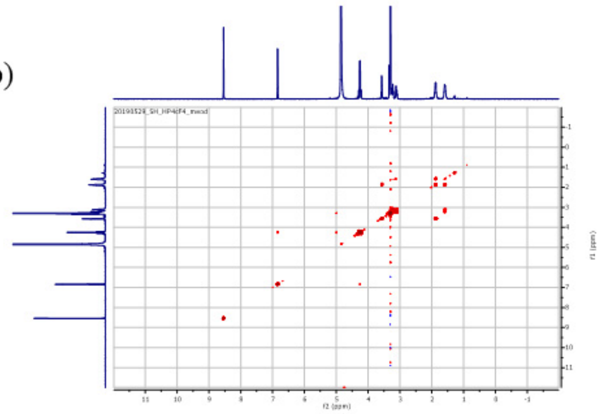

(c)

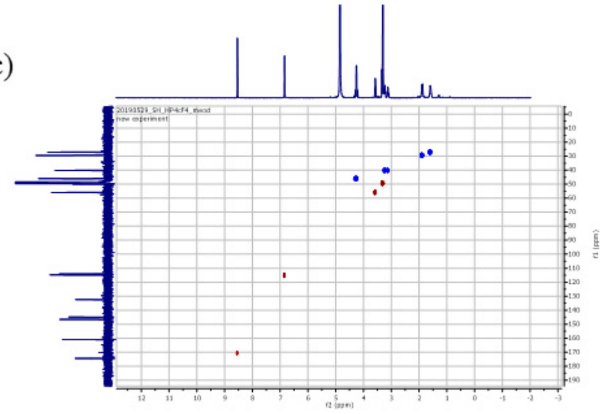

Figure S5. (a) HMBC (**b**) COSY and (**c**) HSQC spectra of the new compound, recorded in MeOD on a 600 MHz NMR instrument.
